# Vulvovaginal candidiasis and antifungal susceptibility pattern of isolated *Candida* spp. among women in Aden Governorate, Yemen

**DOI:** 10.1186/s12879-025-12471-4

**Published:** 2026-01-02

**Authors:** Ali N. M. Gubran, Mohammed Ali Ahmed Al-Baghdadi, Naif Mohammed Al-Haidary

**Affiliations:** 1 Department of Health Sciences, Faculty of Medicine and Health Sciences, University of Science and Technology, Aden, Yemen; 2 Department of Medical Laboratory, Faculty of Medicine and Health Sciences, Aden University, Aden, Yemen; 3 Department of Medical Microbiology and Clinical Immunology, Faculty of Medicine and Health Sciences, Sana’a University, Sana’a, Yemen

**Keywords:** Vulvovaginal candidiasis, *Candida*, Antifungal susceptibility pattern, Aden, Yemen

## Abstract

**Background:**

Vulvovaginal Candidiasis is a complicated inflammatory infectious disease of the female genital tract caused by the genus *Candida*. Most studies dealing with VVC in Yemen did not involve both risk factors and antifungal sensitivity. So, this study was performed to evaluate risk factors contributing to VVC and to evaluate the antifungal susceptibility patterns of *Candida* spp. among women in Aden, Yemen.

**Methods:**

One hundred and two women were enrolled in this cross-sectional study. Two high vaginal swabs were collected, tested microscopically, and inoculated in SDA. A chromogenic medium was used to identify and differentiate *Candida* species. Six available antifungal agents were used for sensitivity testing included: Itraconazole, Fluconazole, Ketoconazole, Clotrimazole, Nystatin and Miconazole by disc diffusion method. The data was finally analyzed by using SPSS software (Version 21).

**Results:**

A total of 102 women were included in this study. The mean age was 27.36 ± 7.7 years, with a range of 16–47 years. The overall frequency of VV among women was 39.2%. The highest rate was 44.9% (22/49) in the age group < 25 years. Five species of *Candida* were isolated, with *C. albicans* being the most frequent (55%), followed by *C. krusei* (17.5%), *C. glabrata* (12.5%), *C. tropicalis* (10%) and *C. parapsilosis* (5%). Symptom severity was significantly associated with VVC (*p* = 0.0001), whereas the association with pregnancy did not reach statistical significance (*p* = 0.066). In terms of antifungal susceptibility testing, the overall resistance was 20% to Clotrimazole, 15% to Nystatin, 7.5% to both Ketoconazole and Miconazole and the lowest resistance rate (2.5%) was to Fluconazole. All five isolated species were 100% sensitive to Itraconazole.

**Conclusion:**

It can be concluded from this study that the overall frequency of VVC among women in Aden-Yemen is higher than that reported among Yemeni women, while slightly lower than that reported globally. The infection was high among women in the age group < 25 years. *C. albicans* and *C. krusei* were the most frequent species. The pregnancy may increase the risk of VVC. Resistance was found to Clotrimazole, Nystatin, Ketoconazole, Miconazole, and Fluconazole, while no resistance was detected to Itraconazole among those species. The resistance among non-albicans *Candida* (NAC) species has increased.

## Background

*Candida* species (*Candida* spp.) are yeast-like fungi that are considered the main causative agents of fungal infections worldwide [1]. They are usually harmless in immunocompetent individuals, where about 20–50% of asymptomatic women have *Candida* spp as flora in their lower genital tract. However, they are considered opportunistic pathogens when the immune system is lowered or mucosal barriers are disrupted. So, they can invade host tissues and cause diseases [2]. They can cause oral and genital candidiasis (thrush), esophagitis, and chronic mucocutaneous candidiasis [3]. Disseminated infections such as Candidemia, endophthalmitis and endocarditis may occur in immunocompromised patients [4].

Vulvovaginal Candidiasis (VVC) is a complicated inflammatory infectious disease of the female genital tract caused by the genus *Candida* [5]. This type of candidiasis is a global health concern due to its association with significant healthcare costs, sexually transmitted diseases (STDs), and ascending vaginal infections [6]. VVC occurs at least once during the lifetime of about 30–50% of women [7]. Numerous risk factors may increase the severity and complications of VVC, including changes in the immune system, elevated vaginal glycogen production, increased estrogen levels, asymptomatic colonization during pregnancy, and pregnancy itself [8].

There are several species belong to genus *Candida* which can be divided into *Candida albicans* and non-albicans *Candida* (NAC). The latter includes *Candida glabrata, Candida krusei, Candida tropicalis, Candida parapsilosis, Candida dubliniensis*, and *Candida lusitaniae.* The majority of candidiasis are caused by *C.albicans* [9, 10].

The diagnosis of VVC is made by direct microscopy and by growing the fungi on Sabouraud’s dextrose agar (SDA) at 30 °C. Along with inoculation in chromogenic media, the species are differentiated by morphologic characteristics on culture media, carbohydrate fermentation, and germ tubes, and the ability of some of them to produce hyphae [11].

The antifungals-resistant species of *Candida* have emerged, leading to systemic candidiasis [12]. Most species appear resistant at least to one or more antifungal drugs (9). Certain NAC species, such as *C. krusei*, are naturally (intrinsically) resistant to fluconazole (FCZ) [13]. Azoles are among the commonly used antifungals that inhibit the fungal cell membrane by interfering with the conversion of lanosterol to ergosterol [3]. Resistance in *Candida* spp. to azoles, such as FCZ, occurs either through efflux pumps that expel the drug from the cell or by modification of the target enzyme (14α-demethylase) responsible for ergosterol production [13]. NAC species also protect themselves against antifungals through biofilm formation [1]. These resistance mechanisms complicate the treatment of *Candida* infections, leading to recurrent fungal infections and severe complications. The increasing antifungal resistance rates represent a burden, necessitating the use of alternative antifungals and the development of new drugs [6, 9].

In Yemen, several studies have detected the prevalence of VVC at 70.0% in Hajjah, 61.5% in Ibb [14, 15], 51.6% in Sana’a, and 20.8% in Aden [16]. Most previous studies focused on determining the prevalence of VVC, while they did not involve both risk factors and antifungal sensitivity. Therefore, this study was conducted to evaluate the risk factors contributing to VVC and to evaluate the antifungal susceptibility patterns of *Candida* spp. among women in Aden, Yemen.

## Methods

### Study design and population

This cross-sectional study was conducted from January 1 to the end of March 2023 at the Gynecology and Obstetrics (G&O) clinic in Al-Sadaqa Teaching Hospital, Aden, Yemen. One hundred and two adult women were involved in the study. The inclusion criteria were women of reproductive age (15–50 years) who had vulvovaginal symptoms such as vaginal discharge, itching, irritation, or discomfort, and who consented to participate in the study. Both pregnant and non-pregnant women were included. The exclusion criteria were women younger than 15 years or older than 50 years, women without vulvovaginal symptoms, those with known immunocompromising conditions (e.g., HIV), and women who had used antifungal or antibiotic treatments within the two weeks prior to sample collection, as these could affect the detection of *Candida* species. Additionally, women with systemic infections or chronic underlying medical conditions that could confound the study outcomes were also excluded.

### Data collection

Previously designed questionnaires with some modifications were used in our research. It included sociodemographic data, such as age, economic status, and level of education. It also gathered clinical data such as severity of symptoms, vaginal discharge, and recurrent infection, as well as questions about risk factors related to VVC infection, including the presence or absence of pregnancy and diabetes. Additionally, the questionnaire included questions on contraceptive use, antibacterial and antifungal treatments, and the direction of genital wiping after toilet use [17].

### Specimen collection

Clinically investigated symptoms and vaginal abnormalities were recorded by gynecologists. Before the collection of samples, the objectives of the study were explained to all participants, who were then asked to provide their consent. Two high vaginal swabs were collected from each participant by a gynecological specialist. Swabs were transported to the laboratory at the Faculty of Medicine & Health Sciences, Aden University, within 30 minutes. The first swab was used for direct microscopy to detect yeast cells or pseudohyphae, while the second swab was used for culture.

**Study procedure**s

These swabs were tested by microscopy and confirmed by inoculation in SDA. Chromogenic medium (HiMedia-M1297A) was used to identify and differentiate the *Candida* species. The inoculated media were incubated at 35 °C for 24–48 hours, and species were identified based on colony morphology and biochemical tests such as carbohydrate fermentation and other tests as the germ tube test for *C. albicans.* Colonies of *Candida albicans* appeared light green on chromogenic media, while other NAC species exhibited distinct colors: *C. krusei* presented with rough, pink colonies, *C. glabrata* appeared as small white colonies, and *C. tropicalis* was identified by its dark blue colonies.

### Diagnostic criteria

Women were considered to have vulvovaginal candidiasis if they had compatible symptoms and either (i) direct microscopy of a vaginal swab (saline/10% KOH wet mount) showed budding yeast and/or pseudohyphae, or (ii) culture on Sabouraud dextrose agar at 30 °C with subsequent subculture on chromogenic medium at 35 °C yielded growth of *Candida* spp. within 24–48 h. Species identification was performed using chromogenic characteristics and conventional tests (germ tube, carbohydrate fermentation). When microscopy and culture were discordant, the culture result was used for species identification and susceptibility reporting.

### Antifungal susceptibility testing

The disc diffusion method for antifungal susceptibility testing was employed as a screening tool using Mueller-Hinton agar supplemented with 2% glucose and 0.5 μg/mL methylene blue. Six available antifungal agents were used for sensitivity testing, including Itraconazole (ICZ, 10 μg), Fluconazole (25 μg), Ketoconazole (KCZ, 15 μg), Clotrimazole (CTZ, 10 μg), Nystatin (NS, 100 IU), and Miconazole (MCZ, 10 μg) (Sigma-Aldrich Quimica, Madrid, Spain). Fluconazole susceptibility was not tested for *C. krusei* due to its intrinsic resistance. The inoculated plates were incubated at 35 °C for 24 hours. Antifungal susceptibility results were documented by measuring the zone of inhibition around the antifungal discs. Resistance was determined based on the diameter of the inhibition zones, which were measured according to Clinical and Laboratory Standards Institute (CLSI) protocol. The breakpoints for antifungal agents used for *Candida* spp. depended on the species, with the following standards: ICZ (S ≥ 23 mm; I = 14–22 mm; R < 13 mm); FCZ (S ≥ 17 mm; I = 14–16 mm; *R* ≤ 13 mm); KCZ (S ≥ 20 mm; I = 10–19 mm; R < 10 mm); CTZ (S ≥ 20 mm; I = 10–19 mm; R < 10 mm); NS (S ≥ 15 mm; I = 10–14 mm; R < 10 mm); and MCZ (S ≥ 22 mm; I = 14–21 mm; *R* ≤ 13 mm) [18].

### Data analysis

The data were analyzed using SPSS software (Version 21). Quantitative data were expressed as mean ± standard deviation (SD) because they followed a normal distribution, while percentages were used to express qualitative data. The association between two or more qualitative variables and the detection of p-values were determined by the chi-square (χ^2^) test. All p-values are two-tailed, and *p* < 0.05 was considered statistically significant.

## Results

A total of 102 women were included in this study. The total mean age ± SD was 27.36 ± 7.7 years, with a range of 16–47 years (Table 1).

**Table 1:** Distribution of studied group according to their ages

Age groups/years	studied group	
	No.	%
< 25	49	48.0
25–34	34	33.3
> 34	19	18.6
Total	102	100

The overall frequency of VVC among women was 39.2% (40/102). The highest rate was 44.9% (22/49) in age group < 25 years (Table 2).

**Table 2:** The frequency of VVC among women according to their ages in Aden, Yemen

Age groups/years	Vulvovaginal candidiasis					
	Positive		Negative		Total	
	No.	%	**No**.	%	No.	%
< 25	22	44.9	27	55.1	49	48.0
25–34	11	32.4	23	67.6	34	33.3
> 34	7	36.8	12	63.2	19	18.6
Total	40	39.2	62	60.8	102	100

**Fig. 1:**
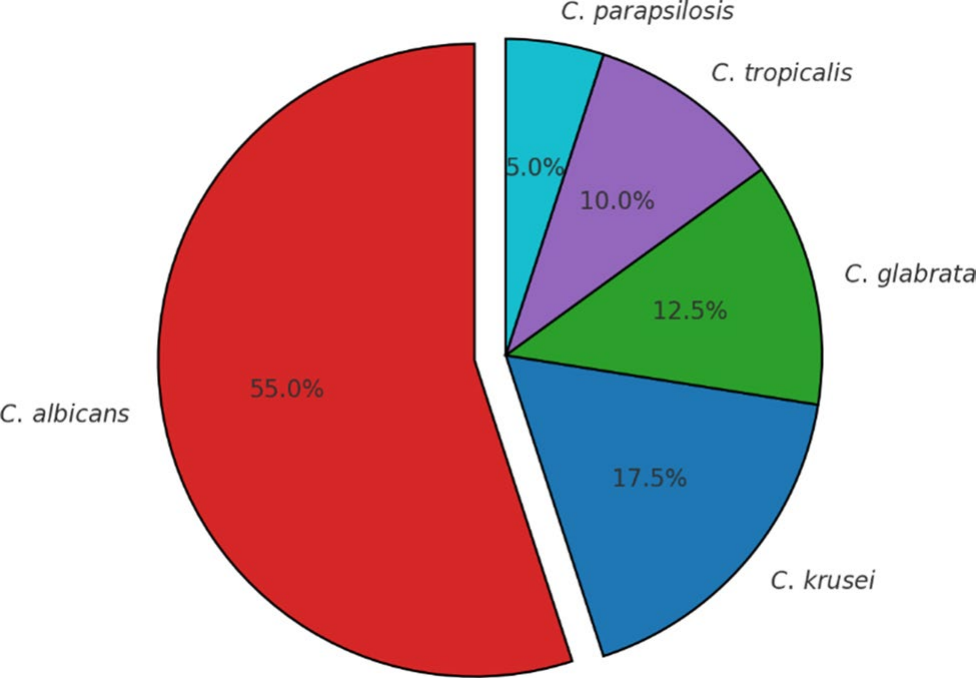
Distribution of *Candida* species isolated from women with vulvovaginal candidiasis in Aden, Yemen

Five species of *Candida* were isolated, with *C. albicans* being the most frequent, found in 55% of the women, followed by *C. krusei* (17.5%), *C. glabrata* (12.5%), *C. tropicalis* (10%), and *C. parapsilosis* (5%) (Fig. 1).

In terms of symptom severity, women with severe symptoms of VVC were more likely to have positive *Candida* cultures compared to those with mild symptoms. Of the 80 women who had severe symptoms, 48.4% (39/80) were diagnosed with VVC, whereas only 4.5% (1/22) of women with mild symptoms had a positive VVC infection. This suggests a significant association between symptom severity and the presence of VVC (*p* = 0.0001). There was a non-statistically significant trend toward an association between VVC and pregnancy (*p* = 0.066) (Table 3).

**Table 3:** Association of severity of the symptoms, vaginal discharge and recurrence of infections and some risk factors with VVC among women in Aden, Yemen

Variable	VVC +ve		p	Variable	VVC +ve		p-value
	No.	%			No.	%	
**Pregnancy**				**Wiping direction in toilet**			
Pregnant (*n* = 38)	19	50.0	0.066	Forward wiping (*n* = 68)	26	38.2	0.469
Non-pregnant (*n* = 64)	21	32.8		Backward wiping (*n* = 34)	14	40.2	
**Symptoms**				**Use antibiotics**			
Severe (*n* = 80)	40	49.4	0.0001	Yes (*n* = 13)	4	30.8	0.364
Mild (*n* = 22)	1	4.5		No (*n* = 89)	36	40.4	
**Recurrent infection**				**Use antifungal**			
Yes (*n* = 57)	22	38.6	0.523	Yes (*n* = 13)	4	30.8	0.364
No (*n* = 45)	18	40.0		No (*n* = 89)	36	40.4	
**Education level**				**Use contraceptive**			
Illiterate (*n* = 13)	2	15.4		Yes (*n* = 5)	1	20.0	0.346
Primary (*n* = 41)	20	48.8	0.656	No (*n* = 97)	39	40.2	
Secondary (*n* = 32)	12	37.5		**Diabetics**			
University(*n* = 16)	6	37.5		Yes (*n* = 1)	1	100.0	0.392
**Income**				No (*n* = 101)	39	38.6	
< 66$(*n* = 64)	25	39.1	0.565				
> 66$(*n* = 38)	15	39.5					
**Vaginal discharge**							
Presence (*n* = 101)	40	39.6	0.608				
Absence (*n* = 1)	0	0.0					

**Fig. 2:**
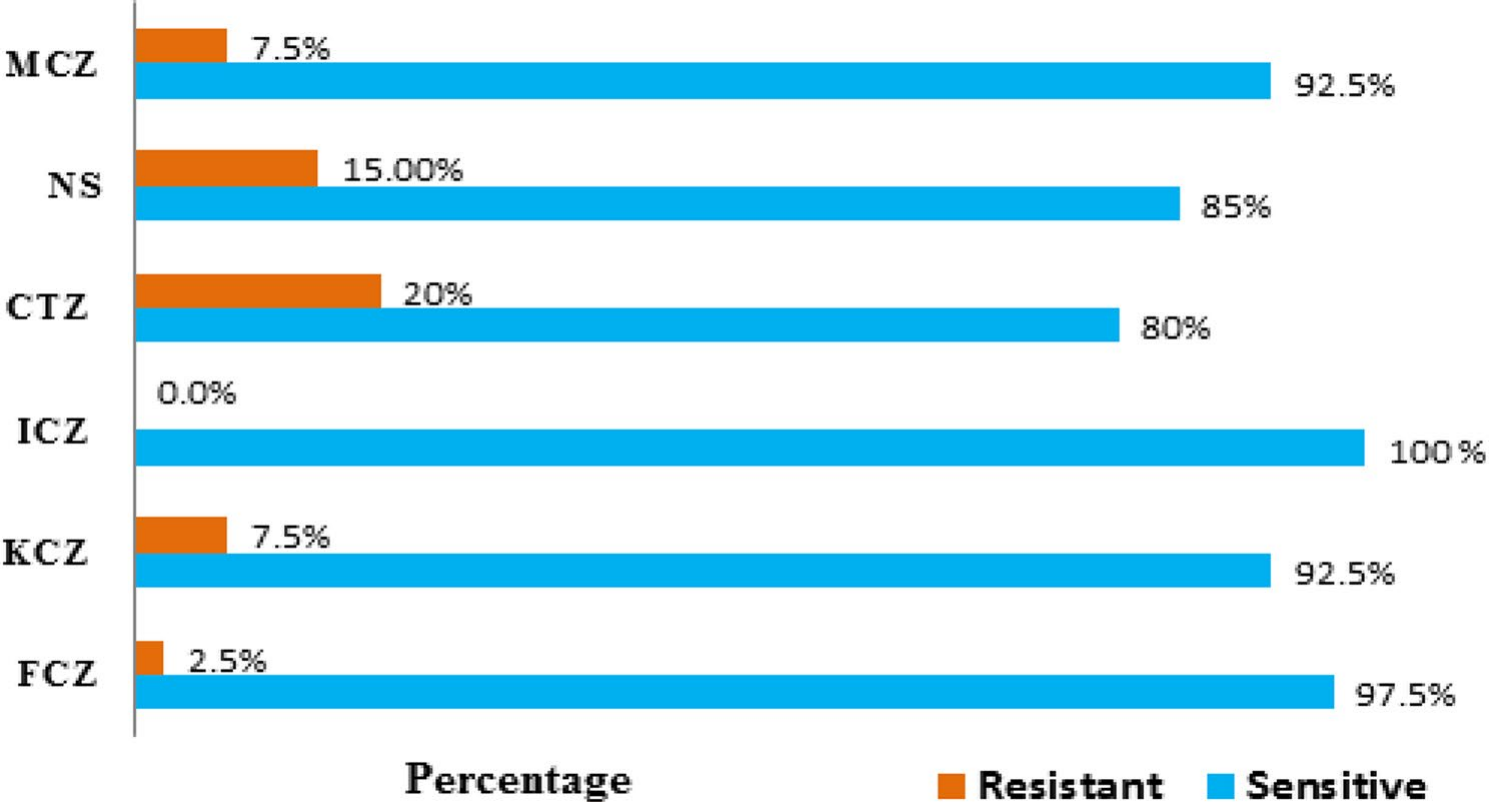
Overall antifungal susceptibility of *Candida* isolates from women with vulvovaginal candidiasis in Aden, Yemen. The horizontal bar chart shows the proportions of isolates categorized as resistant and sensitive to six antifungal agents: fluconazole (FCZ), ketoconazole (KCZ), itraconazole (ICZ), clotrimazole (CTZ), nystatin (ns), and miconazole (MCZ)

The resistance of *Candida* spp. to antifungal drugs was 20% for CTZ, followed by 15% for NS, and 7.5% to each of KCZ and MCZ. The lowest resistance rate was 2.5% for FCZ and no resistance was observed to itraconazole (ICZ, 0%) (Fig. 2).

**Fig. 3:**
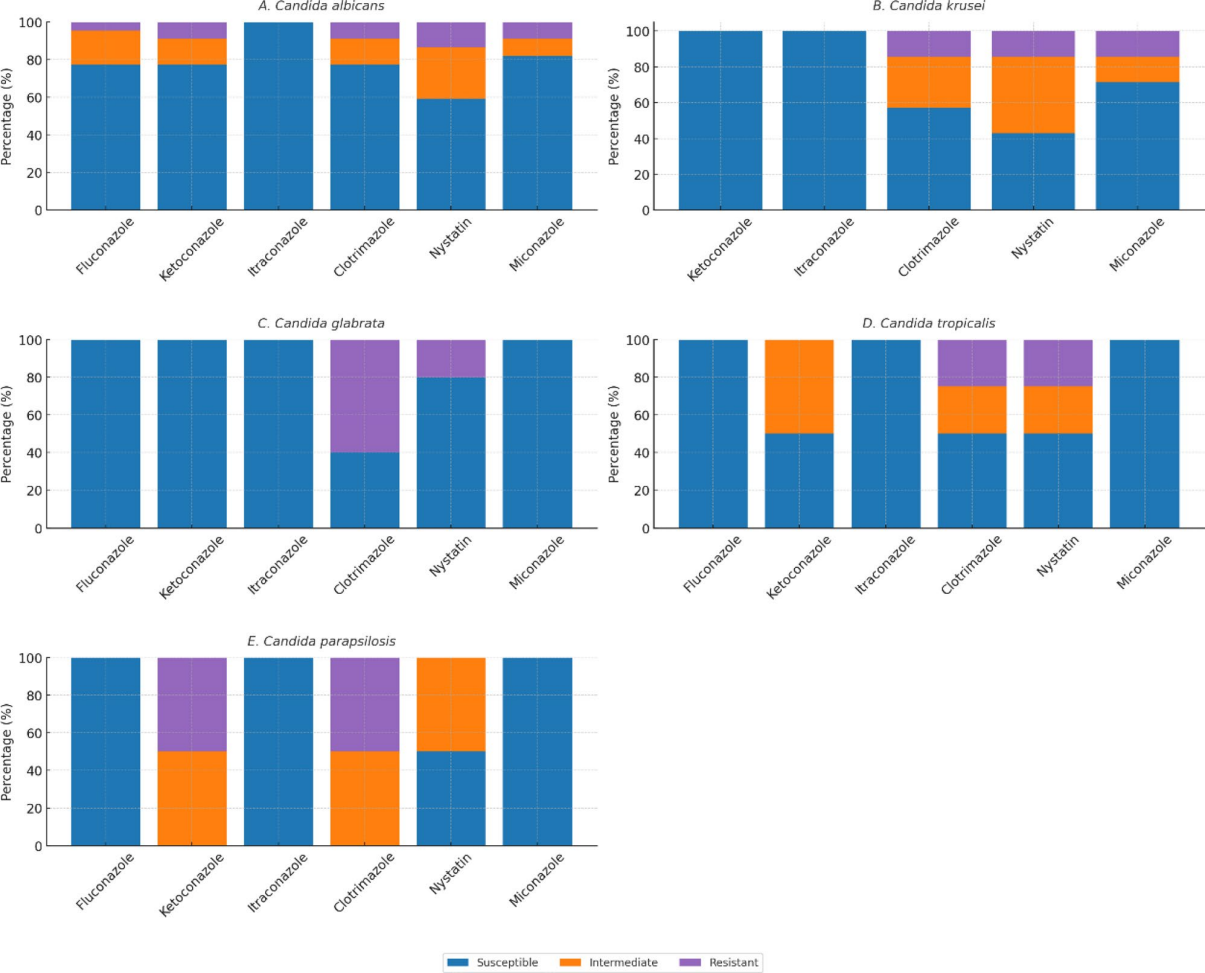
Antifungal susceptibility patterns of *Candida* species isolated from women with vulvovaginal candidiasis in Aden, Yemen. Each panel displays the proportion of isolates categorized as susceptible, intermediate, or resistant to six antifungal agents: fluconazole, ketoconazole, itraconazole, clotrimazole, nystatin, and miconazole. Species-specific responses are shown for: (**A**) *Candida albicans*, (**B**) *Candida krusei* (fluconazole not tested; intrinsic resistance), (**C**) *Candida glabrata,* (**D**) *Candida tropicalis,* and (**E**) *Candida parapsilosis*

According to antifungal resistance rates among *Candida* spp, *C. glabrata* exhibited the strongest resistance to CTZ at a rate of 60%, followed by 50% of *C. parapsilosis.* Additionally, 25% of *C. tropicalis* exhibited resistance to NS, whereas 20% of *C. glabrata* showed resistance. Fifty percent of *C. parapsilosis* exhibited resistance to KCZ and 14.3% of *C. krusei* was resistant to MCZ. Among species tested for fluconazole, all isolates were susceptible except *C. albicans* (4.5%); fluconazole was not tested for *C. krusei* (Fig. 3).

## Discussion

The overall prevalence of VVC was 39.2% in this study. This result is consistent with findings from Iran, where the prevalence was 39.76% [19]. Nearly similar rates were 41.4% and 41.8% noticed in Ethiopia and India [6, 20]. In contrast, other studies revealed higher rates than ours such as 45% in Somalia [21], 50.2% and 50.4% in Egypt [10, 22], 54.66% in Iraq [17], 55% and 61.8% in Iran [19, 23]. One of the highest percentage rates was 84.5% in Nigeria [24]. On the other hand, lower rates were observed such as 5.44% in Brazil [25], 14.0% in Nigeria [26], 22.21% in Saudi Arabia [27], 24% in Libya [28], 28.3% in Iran [29], in Yemen 20.8% [16], 30% in Brazil [30] and 31% in India [31]. These variations could be explained by the personnel behaviors, hygiene, and socioeconomic status of women in different countries [32].

According to the age group, the highest prevalence rate of VVC was 44.9% among women aged < 25 years. A similar finding was shown in a study by Pavani et al., in which women in the age group 18–25 years had the highest VVC rate [33]. Several studies revealed different results as 20–30 years [19, 26, 34–36], 21–25 years [37], 23–26 years [38], 26–30 years [27], 28–37 [14], 25–34 years [16] and 30–34 years [17]. Overgrowth of *Candida* yeasts and increase of VVC among women within this range of age due to an increase of sexual activity among women. In addition to the protection routes that might be used during menstrual loss, wearing of tight clothing, and directions wiping for vagina [39].

In the current study, among five *Candida* species*, C. albicans* was the most commonly isolated, and *C. krusei was* the commonest one of NAC, followed by *C. glabrata*, *C. tropicalis and C. parapsilosis*. Bitew & Abebaw, found similar observations where *C. albicans* and *C. krusei* were predominant among *C. albicans* and non-*albicans* species respectively [6]. On the contrary, the finding was recorded by Ghaddar et al. and Waikhom et al. where *C. glabrata* was the most isolated yeast, followed by *C. albicans* [40, 41]. Different reports were recorded by other authors as *C. albicans* and *C. glabrata* [17, 21, 29, 42–48], C. *albicans* and *C. tropicalis* [23, 31, 49], *C. albicans, C. tropicalis* and *C. krusei* [9]*, C. albicans* and *C. lusitaniae* [10], *C. albicans* and *C. parapsilosis* [50] and *C. albicans* and *C. lusitaniae* [51]. The sensitivity of techniques used for identifying the *Candida* species and the different geographical distribution of those species among those populations could be explained by the differences in global distribution [52, 53].

About the severity of the symptoms and risk factor, there was a significant association between the VVC and severe symptoms (*p* = 0.0001). The association was also significant between VVC and pregnancy (*p* = 0.066). Al-akeel et al. and Abdullah showed a significant association between pregnancy and positive culture (*p* = 0.047) [17, 27]. Siddiqui reported different data [54]. Hormonal variations may contribute to increasing VVC among pregnant women [32].

In terms of susceptibility testing for antifungal drugs in the present data, the overall resistance among *Candida* spp. was 20% of CTZ (10 µg), followed by 15%NS (100 IU), 7.5% to KCZ (15 µg) and MCZ (10 µg) each and lowest rate of resistance was 2.5% for FCZ (25 µg). Three researchers reported that resistance to CTZ was 24.2%, 24.6%, and 59.3% [9, 14, 55].

According to the present findings, the highest resistance rate to CTZ among *Candida* spp. was for C*. glabrata* 60%, followed by C. *parapsilosis* 50%. Slightly similar results were reported by Khan et al. who showed that 62.5% of C*. glabrata* was resistant to CTZ [9]. Deorukhkar and Saini reported that 50% of *C. parapsilosis* to CTZ [56]. In contrast, Das et al., found that 50% of *C. krusei* was CTZ resistant [55].

Khan et al. reported that the resistance rate to NS was 58.3% [9]. Two studies revealed that 9.3% of isolates were NS resistant [41, 50]. Lower rate was noticed by Edrees et al. where the resistance rate of those yeasts to NS was 5.2% [14]. In the present results, 25% *C. tropicalis* was resistant to NS, followed by 20% *C. glabrata*. A study by Khan et al. found that 68.7% of *C. glabrata* and 38.8% *C. tropicalis* were resistant to NS [9]. Other studies showed that there were no isolates resistant to NS [45, 57].

Two studies from Yemen and India recorded that 26.87% and 35.3% of isolates were KCZ resistant, respectively [14, 20]. A lower percentage (3.4%) was detected in Brazil [30], while no KCZ resistant isolates were reported in Saudi Arabia and India [27, 45]. In the obtained data, 50% of *C. parapsilosis* was resistant to KCZ. Different rate was reported by Das *et al.,* in which 30% of *C. krusei* was resistant to KCZ [55]. No KCZ resistant isolates were reported in Saudi Arabia and India [27, 45].

Researcher from Vietnam observed that 13.0% of isolates were resistant to MCZ [50]. Another study showed that 63% of isolates were MCZ resistant [56]. Our findings revealed that 14.3% of *C. krusei* was resistant to MCZ. Sasikala & Udayasri detected no resistance to MCZ among all isolates [45].

Somewhat similar to our data, Dharmik et al. detected that 2.8% of *Candida* species were resistant to FCZ [56]. Goulart et al. showed a lower rate of resistance, which was 1.7% [30]. Several studies demonstrated different percentages of resistance rates to FCZ such as 8.7% [50], 8.96% [14], 15.6% [55], 17.2% [6], 25% [54], 42% [58], and 62% [9]. A study in Iran found, there was not any isolate resistant to FCZ [29]. All species were sensitive to FCZ except *C. albicans*, where the rate of resistance was 4.5% in this study. Al-akeel et al. revealed that 5% of *C. albicans* was FCZ resistant [27]. A study in Egypt found that 3.1% of *C. albicans* was resistant [10]. Bitew and Abebaw found that 2% of *C. albicans* was resistant to FCZ [6]. Two studies from Iran and Kuwait revealed that all species were 100% sensitive to FCZ [29, 42]. Different findings were noticed by Deorukhkar and Saini, where the resistance to FCZ among *Candida* spp. were 29.5%, 27.3, and 25% for *C. tropicalis*, *C. glabrata* and *C. kefyr*, respectively [56]. Other studies showed that 71.43, and 100% of *C. krusei* were resistant to FCZ [9, 45, 57]. *C. krusei* was naturally resistant to FCZ [13].

In the current study, all five isolated species were 100% sensitive to ICZ (10 µg). This contradicts the studies from different regions globally [9, 14, 46, 48, 58, 59]. Our study noticed that the NAC is more resistant to most antifungal agents than *C. albicans*. The formation of biofilm plays a role in the difference in the ability of *Candida* species to resist antifungal drugs [60].

Although this study was conducted in a specific geographic area, the findings provide valuable insight into antifungal resistance patterns in a resource-limited setting. *Candida* species distribution and drug susceptibility vary significantly by region, and data from underrepresented populations such as Yemeni women are essential to global surveillance efforts. Reporting local resistance patterns contributes to the broader understanding of emerging antifungal resistance and supports evidence-based strategies for diagnosis and treatment, both regionally and internationally.

## Limitations of the study

This study has several limitations. First, the sample size was relatively small, which may limit the generalizability of the findings to a broader population. A larger sample size would provide more robust data on the prevalence of VVC and antifungal resistance patterns. Second, this study relied on conventional methods, including chromogenic agar, for preliminary identification of *Candida* spp., which cannot definitively distinguish all species. More advanced techniques, such as polymerase chain reaction (PCR), would improve species-level accuracy and diagnostic precision. Third, the cross-sectional design of the study limits the ability to establish causality between risk factors and the development of VVC. Fourth, although antifungal susceptibility testing was conducted using standardized disc diffusion techniques, the CLSI broth microdilution method (M27–A3/S4) was not utilized due to resource constraints. This method is considered the gold standard and may offer more precise and reproducible results. Future studies should incorporate this approach where feasible to strengthen susceptibility data. Finally, the study did not investigate the clinical outcomes of treatment, which would provide valuable insights into the effectiveness of antifungal therapy in this population.

## Conclusions

It can be concluded from this study that the overall frequency of VVC among women in Aden, Yemen, is higher than that reported among Yemeni women, while it is slightly lower than most reported globally. The infection was high among women in the age group < 25 years. *C. albicans* and *C. krusei* were the most frequent species. Pregnancy can increase the risk of VVC. Among the five antifungal agents, resistance was found against CTZ, NS, KCZ, MCZ, and FCZ, while no resistance to ICZ was detected among those species. The resistance has increased among NAC species.

## Data Availability

This article included all necessary data and if there is further data needed, it can be made available by the authors at any time.

## Abbreviations

*Candida* spp: *Candida* species
VVC: Vulvovaginal Candidiasis
STDs: Sexually transmitted diseases
*NAC*: Non-albicans *Candida*
*C. albicans*: *Candida albicans*
*C. glabrata*: *Candida glabrata*
*C. krusei*: *Candida krusei*
*C. tropicalis*: *Candida tropicalis*
*C. parapsilosis*: *Candida parapsilosis*
*C. dubliniensis*: *Candida dubliniensis*
*C. lusitaniae*: *Candida lusitaniae*
G&O: Gynecology & Obstetrics
SDA: Sabouraud’s dextrose agar
FCZ: 25 µg: Fluconazole
KCZ: 15 µg: Ketoconazole
CTZ: 10 µg: Clotrimazole
ICZ: 10 µg: Itraconazole
NS: 100 IU: Nystatin
MCZ: 10 µg: Miconazole
SD: Standard deviation
χ2: Chi-square
CLSI: Clinical and Laboratory Standards Institute
NA: Not available
Fig: Figure
n: number
S: Sensitive
I: Intermediate
R: Resistant

## Declarations

### Author information

A.N.M.G is an Assistant Professor in Diagnostic and Molecular Microbiology Department of Health Sciences, Faculty of Medicine and Health Sciences, University of Science and Technology, Aden, Yemen, M.A.A.A is an Associate Professor in Microbiology and Immunology Department of Medical Laboratory, Faculty of Medicine and Health Sciences, Aden University, Aden, Yemen, N.M.A has an MSC degree in Medical Microbiology, Department of Medical Microbiology and Clinical Immunology, Faculty of Medicine and Health Sciences, Sana’a University, Sana’a, Yemen.

## References

[1] Gómez-Gaviria M, Mora-Montes HM. Current aspects in the biology, pathogeny, and treatment of *Candida krusei*, a neglected fungal pathogen. Infect Drug Resist. 2020;10:1673–89. 10.2147/IDR.S247944 PMC7293913 32606818

[2] Ahmed N, Mahmood MS, Ullah MA, Araf Y, Rahaman TI, Moin AT, et al. COVID-19-Associated candidiasis: possible patho-mechanism, predisposing factors, and prevention strategies. Curr Microbiol. 2022;79(5):127. 35287179 10.1007/s00284-022-02824-6 PMC8918595

[3] Levinson W, Chin-Hong P, Joyce E, Nussbaum J, Schwartz B. Review of Medical Microbiology and Immunology: a Guide to Clinical infectious diseases. 13th. New York: McGraw-Hill Education; 2018.

[4] Gajdzis M, Figuła K, Kamińska J, Kaczmarek R. Endogenous endophthalmitis-the clinical significance of the primary source of infection. J Clin Med. 2022;11(5):1183. 35268274 10.3390/jcm11051183 PMC8911070

[5] Willems HME, Ahmed SS, Liu J, Xu Z, Peters BM. Vulvovaginal candidiasis: a Current understanding and burning questions. J Fungi (Basel). 2020;6(1):27. 32106438 10.3390/jof6010027 PMC7151053

[6] Bitew A, Abebaw Y. Vulvovaginal candidiasis: species distribution of *Candida* and their antifungal susceptibility pattern. BMC Women’s Health. 2018;18:1–10. 29902998 10.1186/s12905-018-0607-z PMC6003188

[7] Otoo-Annan E, Senoo-Dogbey VE. Recurrent vulvovaginal candidiasis: assessing the relationship between feminine/vaginal washes and other factors among Ghanaian women. BMC Public Health. 2024;24(1):100. 38183091 10.1186/s12889-024-17668-x PMC10768209

[8] Gonçalves B, Ferreira C, Alves CT, Henriques M, Azeredo J, Silva S. Vulvovaginal candidiasis: epidemiology, microbiology and risk factors. Crit Rev In Microbiol. 2016;42(6):905–27. 26690853 10.3109/1040841X.2015.1091805

[9] Khan M, Ahmed J, Gul A, Ikram A, Lalani FK. Antifungal susceptibility testing of vulvovaginal *Candida* species among women attending antenatal clinic in tertiary care hospitals of Peshawar. Infect Drug Resist. 2018;3:447–56. 10.2147/IDR.S153116 PMC5878663 29628769

[10] Shawaky SM, Al Shammari MM, Sewelliam MS, Ghazal AA, Amer AN. A study on vaginitis among pregnant and non-pregnant females in Alexandria, Egypt: an unexpected high rate of mixed vaginal infection. Aims Microbiol. 2022;8(2):167. 35974993 10.3934/microbiol.2022014 PMC9329880

[11] Maikan HK, Jabbar S, Al-Haishawi H. Isolation and identification of *Candida tropicalis* as a cause of cutaneous candidiasis in Kalar District, Iraq. Arch Razi Inst. 2022;77(4):1377–82. 36883146 10.22092/ARI.2022.357613.2066 PMC9985788

[12] Lindberg E, Hammarström H, Ataollahy N, Kondori N. Species distribution and antifungal drug susceptibilities of yeasts isolated from the blood samples of patients with candidemia. Sci Rep. 2019;9(1):3838. 30846717 10.1038/s41598-019-40280-8 PMC6405987

[13] Chowdhary A, Prakash A, Sharma C, Kordalewska M, Kumar A, Sarma S, et al. A multicentre study of antifungal susceptibility patterns among 350 *Candida auris* isolates (2009-17) in India: role of the ERG11 and FKS1 genes in azole and echinocandin resistance. J Antimicrob Chemother. 2018;73(4):891–99. 29325167 10.1093/jac/dkx480

[14] Edrees WH, Al-Asbahi AA, Al-Shehari WA, Qasem EA. Vulvovaginal candidiasis prevalence among pregnant women in different hospitals in Ibb, Yemen. Univers J Pharm Res. 2020;15.

[15] Edrees W, Al-Ofairi B, Alshwmi M, Al-Awar MS. Prevalence and antifungal susceptibility of *Candida* species causing vaginitis among pregnant women in‎ Hajjah Governorate, Yemen. Al-Razi Univ J for Med Sci. 2021;5(2).

[16] Binsaad AJ, Al-Abd N. The prevalence of vulvovaginal candidiasis (VVC) among women suffering vaginitis attended a private gynecological clinic, Aden-Yemen. Electron J Univ Aden Basic Appl Sci. 2021;2(4):169–75.

[17] Abdullah SM. Prevalence of *Candida* spp. From in women with vulvovaginal infection in maternity teaching hospital in Erbil, Iraq. Iraq Med J. 2020, 26;4(1).

[18] CLSI. Method for antifungal disk diffusion susceptibility testing of yeasts, (M44-ED3). 3rd. Wayne, PA, USA: Clinical and Laboratory Standards Institute; 2018.

[19] Kiasat N, Rezaei-Matehkolaei A, Mahmoudabadi AZ, Mohamadpour KH, Molavi S, Khoshayand N. Prevalence of vulvovaginal candidiasis in Ahvaz, southwest Iran: a semi-large scale study. Jundishapur J Microbiol. 2019, 31;12(3).

[20] Bashir G, Altaf I, Khurshid R, Ahmed T, Ali A, Zaffar S. Identification and pattern of antifungal susceptibility of *Candida* species isolated from cases of vaginitis in a tertiary care hospital in India. Iran J Microbiol. 2023;15(2):318. 37193233 10.18502/ijm.v15i2.12484 PMC10183080

[21] Al-Mamari A. Determining the prevalence of bacterial vaginosis & vulvovaginal candidiasis among married and unmarried women & evaluating the association socio-demographic risk factors & symptoms-related variables in women attending gynecology clinic in Hargeisa group hospital, Hargeisa city, Somaliland. Open J Med Microbiol. 2020;10(3):114–28.

[22] ElFeky DS, Gohar NM, El-Seidi EA, Ezzat MM, AboElew SH. Species identification and antifungal susceptibility pattern of *Candida* isolates in cases of vulvovaginal candidiasis. Alexandria J Med. 2016;52(3):269–77.

[23] Jannati B, Pourdad A, Izadjoo A, Zarrinfar H, Najafzadeh MJ, Fata A. The Prevalence of non-albicans *Candida* and *Candida* Mixed-species in vulvovaginal candidiasis in northeast Iran. Clin Exp Obstet Gynecology. 2024;51(3):77.

[24] Ugwa EA. Vulvovaginal candidiasis in Aminu Kano Teaching Hospital, NorthWest Nigeria: hospital-based epidemiological study. Ann Med Health Sci Res. 2015;5(4):274–78. 26229716 10.4103/2141-9248.160185 PMC4512120

[25] Lopes PH, Pacini VL, Norberg AN. Genital infection by gardnerella vaginalis and *Candida* spp. Among women in Nova Iguacu city, Rio de Janeiro province, Brazil. Open Access Lib J. 2017;4(3):1–7.

[26] Emeribe AU, Nasir IA, Onyia J, Ifunanya AL. Prevalence of vulvovaginal candidiasis among nonpregnant women attending a tertiary health care facility in Abuja, Nigeria. Res Rep In Trop Med. 2015, 29;6:37–42.

[27] Al-Akeel RA, El-Kersh TA, Al-Sheikh YA, Al-Ahmadey ZZ. Prevalence and comparison for detection methods of *Candida* species in vaginal specimens from pregnant and non pregnant Saudi women. Afr J Microbiol Res. 2013;1:56–65.

[28] Altayyar IA, Alsanosi AS, Osman NA. Prevalence of vaginal candidiasis among pregnant women attending different gynecological clinic at south Libya. Eur J Exp Biol. 2016;6(3):25–29.

[29] Rezaei-Matehkolaei A, Shafiei S, Zarei-Mahmoudabadi A. Isolation, molecular identification, and antifungal susceptibility profiles of vaginal isolates of *Candida* species. Iran J Microbiol. 2016;8(6):410. 28491253 PMC5420397

[30] Goulart LS, Santiago EF, Ramon JL, Moura SV, Silva AR, Silva IF Jr, et al. Species distribution and antifungal susceptibility to vulvovaginal Candida spp. In southern Mato grosso State, Brazil. Jornal Brasileiro de Patologia e Medicina Laboratorial. 2016;52:233–37.

[31] Kalia N, Singh J, Sharma S, Kamboj SS, Arora H, Kaur M. Prevalence of vulvovaginal infections and species specific distribution of vulvovaginal candidiasis in married women of north India. Int J Curr Microbiol App Sci. 2015;4(8):253–66.

[32] Disha T, Haque F. Prevalence and risk factors of vulvovaginal candidosis during pregnancy: a review. Infectious diseases in obstetrics and gynecology. 2022;2022(1):6195712. 10.1155/2022/6195712 PMC9329029 35910510

[33] Pavani P, Lavanya V, Kailasanatha Reddy B. Prevalence of vulvovaginal candidiasis and its correlation with gestational age and parity in pregnant women at a tertiary care hospital in south India. IJAR. 2018;4(10):428–31.

[34] Nurat AA, Babalola GO, Shittu MO, Tijani MA, Adekola SA. Detection and epidemiology of vulvovaginal candidiasis among asymptomatic pregnant women attending a tertiary hospital in Ogbomoso, Nigeria. Int J Biomed Res. 2015;6(7):518–23.

[35] Al-Rukeimi AA, Al-Hatami SM, Al-Danany DA, Al-Shamahy HA, Al Rukeimi RA. Prevalence and risk factors associated with vulvovaginal candidiasis during pregnancy in Sana’a, Yemen. Univers J Pharm Res. 2020;15.

[36] Al-Janabi AA, Nama ZF. Impact of vulvovaginal candidiasis on parity and number of living children in pregnant and non-pregnant women. J Xiangya Med. 2024;12.

[37] Kamya Ramesh Swaminathan DM, Gerald S, Swathi C. Prevalence of vulvovaginal candidiasis in the women of the reproductive age, in rural India. Diabetes. 2017;7:5–8.

[38] Jasim ST. The relationship between vulvovaginal candidiasis and some predisposing factors in prevalence among Baghdad women. Sys Rev Pharm. 2020;11(12):1318–22.

[39] Edem EN, Mbong EO, Olaniyan UO. Environmental and human behavioral factors associated with vulvovaginal candidiasis among single and married women in eket. Global J Infect Dis Clin Res. 2021;7(1):037–42.

[40] Ghaddar N, El Roz A, Ghssein G, Ibrahim JN. Emergence of vulvovaginal candidiasis among Lebanese pregnant women: prevalence, risk factors, and species distribution. Infect Dis In Obstet Gynecology. 2019;2019(1):5016810. 10.1155/2019/5016810 PMC6699268 31467477

[41] Waikhom SD, Afeke I, Kwawu GS, Mbroh HK, Osei GY, Louis B, et al. Prevalence of vulvovaginal candidiasis among pregnant women in the Ho municipality, Ghana: species identification and antifungal susceptibility of *Candida* isolates. BMC Pregnancy Childbirth. 2020;20:1–4. 10.1186/s12884-020-02963-3 PMC7201979 32375724

[42] Alfouzan W, Dhar R, Ashkanani H, Gupta M, Rachel C, Khan ZU. Species spectrum and antifungal susceptibility profile of vaginal isolates of *Candida* in Kuwait. J de mycologie medicale. 2015;25(1):23–28. 10.1016/j.mycmed.2014.10.021 25534676

[43] Hasanvand S, Qomi HA, Kord M, Didehdar M. Molecular epidemiology and in vitro antifungal susceptibility of *Candida* isolates from women with vulvovaginal candidiasis in northern cities of Khuzestan province, Iran. Jundishapur J Microbiol. 2017, 31;10(8).

[44] Sangaré I, Sirima C, Bamba S, Zida A, Cissé M, Bazié WW, et al. Prevalence of vulvovaginal candidiasis in pregnancy at three health centers in Burkina Faso. J de mycologie medicale. 2018;28(1):186–92. 10.1016/j.mycmed.2017.08.006 28939305

[45] Sasikala G, Udayasri B. Speciation and antifungal susceptibility profiles of *Candida* isolates from vaginitis patients attending std clinic at a tertiary care hospital. J Dr. YSR Univ Health Sci. 2018;7(2):94–97.

[46] Ghaddar N, Anastasiadis E, Halimeh R, Ghaddar A, Dhar R, AlFouzan W, et al. Prevalence and antifungal susceptibility of *Candida* albicans causing vaginal discharge among pregnant women in Lebanon. BMC Infect Dis. 2020;20:1–9. 10.1186/s12879-019-4736-2 PMC6958632 31931738

[47] Intra J, Sala MR, Brambilla P, Carcione D, Leoni V. Prevalence and species distribution of microorganisms isolated among non-pregnant women affected by vulvovaginal candidiasis: a retrospective study over a 20 year-period. J Med Mycology. Aug 1, 2022;32(3):101278. 10.1016/j.mycmed.2022.101278 35523109

[48] Rachel R, Anuradha M, Leela KV. Biofilm formation and antifungal susceptibility profile of *Candida* species responsible for vulvovaginal candidiasis in pregnant and non-pregnant women visiting a tertiary care hospital in southern India. J Pure Appl Microbiol. 2024;18(1).

[49] Venugopal D, Husain K, Mustafa SA, Sabeen S. Epidemiology, risk factors and antimicrobial profile of vulvovaginal candidiasis (VVC): a study among women in the central region of Saudi Arabia. J Med Mycology. 2021;31(2):101049. 10.1016/j.mycmed.2020.101049 33153879

[50] Anh DN, Hung DN, Tien TV, Dinh VN, Son VT, Luong NV, et al. Prevalence, species distribution and antifungal susceptibility of *Candida* albicans causing vaginal discharge among symptomatic non-pregnant women of reproductive age at a tertiary care hospital. Vietnam. BMC Infect Dis. 2021;21(1):523. 34082699 10.1186/s12879-021-06192-7 PMC8176683

[51] Hashemi SE, Shokohi T, Abastabar M, Aslani N, Ghadamzadeh M, Haghani I. Species distribution and susceptibility profiles of *Candida* species isolated from vulvovaginal candidiasis, emergence of C. lusitaniae. Curr Med Mycology. 2019;5(4):26. 10.18502/cmm.5.4.2062 PMC7034787 32104741

[52] Seyoum E, Bitew A, Mihret A. Distribution of *Candida* albicans and non-albicans *Candida* species isolated in different clinical samples and their in vitro antifungal suscetibity profile in Ethiopia. BMC Infect Dis. 2020;20:1–9. 10.1186/s12879-020-4883-5 PMC7081544 32188422

[53] Bilal H, Shafiq M, Hou B, Islam R, Khan MN, Khan RU, et al. Distribution and antifungal susceptibility pattern of *Candida* species from mainland China: a systematic analysis. Virulence. 2022;13(1):1573–89. 36120738 10.1080/21505594.2022.2123325 PMC9487756

[54] Siddiqui R. Comparison of species distribution, antifungal susceptibility and virulence factors in pregnant and non-pregnant women with vulvo-vaginal candidiasis. J Krishna Inst Of Med Sci (JKIMSU). 2021;10(4).

[55] Das KH, Mangayarkarasi V, Sen M. Antifungal resistant in non-albicans *Candida* species are emerging as a threat to antenatal women with vulvovaginal candidiasis. Biomed Pharmacol J. 2019, Sep, 25;12(2):1369–78.

[56] Dharmik PG, Gomashe AV, Upadhyay VG. Susceptibility pattern of various azoles against *Candida* species causing vulvovaginal candidiasis. J Obstet Gynaecol India. 2013;63(2):135–37. 24431621 10.1007/s13224-012-0280-3 PMC3664691

[57] Emam SM, Elazm AA, Morad AW. Exoenzymes production and antifungal susceptibility of *Candida* species isolated from pregnant women with vulvovaginitis. J Am Sci. 2012;8(12):1392–99.

[58] Brandolt TM, Klafke GB, Gonçalves CV, Bitencourt LR, Martinez AM, Mendes JF, et al. Prevalence of *Candida* spp. In cervical-vaginal samples and the in vitro susceptibility of isolates. Braz J Microbiol. 2017;48(1):145–50. 27756539 10.1016/j.bjm.2016.09.006 PMC5220630

[59] Deorukhkar SC, Saini S. Vulvovaginal candidiasis due to non albicans Candida: its species distribution and antifungal susceptibility profile. Int J Curr Microbiol App Sci. 2013;2(12):323–28.

[60] Rodrigues CF, Silva S, Henriques M. *Candida* glabrata: a review of its features and resistance. Eur J Clin Microbiol Infect Dis. 2014;33:673–88. 24249283 10.1007/s10096-013-2009-3

